# Evidence for chemical interference effect of an allelopathic plant on neighboring plant species: A field study

**DOI:** 10.1371/journal.pone.0193421

**Published:** 2018-02-23

**Authors:** Antonio I. Arroyo, Yolanda Pueyo, M. Luz Giner, Ana Foronda, Pedro Sanchez-Navarrete, Hugo Saiz, Concepción L. Alados

**Affiliations:** 1 Instituto Pirenaico de Ecología (CSIC), Zaragoza, Spain; 2 Departamento de Biología y Geología, Física y Química Inorgánica, Universidad Rey Juan Carlos, Móstoles, Spain; Agroecological Institute, CHINA

## Abstract

Many studies have reported the phytotoxicity of allelopathic compounds under controlled conditions. However, more field studies are required to provide realistic evidences for the significance of allelopathic interference in natural communities. We conducted a 2-years field experiment in a semiarid plant community (NE Spain). Specifically, we planted juvenile individuals and sowed seeds of *Salsola vermiculata* L., *Lygeum spartum* L. and *Artemisia herba-alba* Asso. (three co-dominant species in the community) beneath adult individuals of the allelopathic shrub *A*. *herba-alba*, and assessed the growth, vitality, seed germination and seedling survival of those target species with and without the presence of chemical interference by the incorporation of activated carbon (AC) to the soil. In addition, juveniles and seeds of the same three target species were planted and sown beneath the canopy of adults of *S*. *vermiculata* (a shrub similar to *A*. *herba-alba*, but non-allelopathic) and in open bare soil to evaluate whether the allelopathic activity of *A*. *herba-alba* modulates the net outcome of its interactions with neighboring plants under contrasting abiotic stress conditions. We found that vitality of *A*. *herba-alba* juveniles was enhanced beneath *A*. *herba-alba* individuals when AC was present. Furthermore, we found that the interaction outcome in *A*. *herba-alba* microsite was neutral, whereas a positive outcome was found for *S*. *vermiculata* microsite, suggesting that allelopathy may limit the potential facilitative effects of the enhanced microclimatic conditions in *A*. *herba-alba* microsite. Yet, *L*. *spartum* juveniles were facilitated in *A*. *herba-alba* microsite. The interaction outcome in *A*. *herba-alba* microsite was positive under conditions of very high abiotic stress, indicating that facilitative interactions predominated over the interference of allelopathic plants under those conditions. These results highlight that laboratory studies can overestimate the significance of allelopathy in nature, and consequently, results obtained under controlled conditions should be interpreted carefully.

## Introduction

The composition and structure of plant communities in arid and semiarid environments are markedly influenced by biotic interactions that occur among plants [1,2]. These interactions can be classified as either positive or negative. Positive interactions occur when the presence of one plant (*e*.*g*. a nurse shrub) facilitates the establishment, growth or reproduction of neighboring plants, usually by modifying and improving the microclimatic conditions and resource availability for those plants [3,4]. Negative interactions occur when one plant hinders the presence of neighboring plants. Negative interactions include competition for limiting resources, for instance water and nutrients [1], but also include allelopathy, which is a chemically mediated interaction that has received much less attention.

Allelopathy can be defined as the interference that a plant exerts over other plants through the production and release of “toxic” compounds into the local environment as a result of volatilization, root exudates, leaf leachates and plant litter decomposition [5]. These compounds can directly reduce germination, growth and survival of neighboring plants because of its biological effects on cell division, membrane permeability, respiration or photosynthesis [6], but also indirectly by modifying the activity of associated soil microorganisms and their influence on ecosystem processes [7,8]. Allelopathic plants can cause a number of changes in vegetation patterning including bare rings, inhibition zones, monocultures and root segregation [9,10]. In addition, it has been reported that in species-rich Mediterranean shrublands the presence of an allelopathic shrub can lead to an increase in plant diversity through its negative effects on highly competitive plant species (indirect facilitation) [11]. Therefore, allelopathic interference should be considered as an important driver of community structure in arid and semiarid plant communities.

The net outcome of positive and negative interactions may vary according to the degree of environmental harshness. The frequency and intensity of positive interactions can increase with the level of abiotic stress [12,13] (but see [14]). However, the synthesis, release, accumulation and phytotoxicity of allelopathic compounds may also be enhanced under harsh abiotic conditions, including water scarcity, high temperatures or intense solar radiation [15–17]. Thus, the potential benefits of the more benign microclimatic conditions that occur beneath allelopathic plants may be reduced or negated under more severe abiotic conditions. On the other hand, co-evolution may have led to local adaptation of some species to their chemical neighbor [18,19]. Therefore, a net positive outcome from such interactions might be possible for tolerant species. Despite these processes operating simultaneously, the role of chemical interference modulating the net interaction outcome for allelopathic shrubs in arid and semiarid environments has been largely unexplored.

Several laboratory and greenhouse studies have confirmed the phytotoxicity of chemicals released from allelopathic plants, and this has usually been taken as direct evidence of allelopathy. However, experimental conditions (*i*.*e*. types, concentrations and exposure to allelopathic compounds, and the absence of soil and/or its microorganisms) are far from those found in nature, and consequently, field experiments are needed to provide a realistic understanding of the significance of allelopathy under natural conditions [20]. However, methods required to assess allelopathic interference under natural conditions are challenging [21,22]. Activated carbon (AC) is commonly used as an effective way to reduce the chemical interference of allelopathic plants, because of its large capacity to adsorb biochemical compounds [22–24]. Yet, AC may alter nutrient concentrations in the soil or modify other soil characteristics such as pH and water retention [22,25,26]. These effects can confuse interpretation of the beneficial effects of the release of chemical interference, and this possibility needs to be carefully assessed [25]. Furthermore, most of the few field studies on allelopathy have been performed in managed ecosystems (*e*.*g*. [27–29]) or communities invaded by exotic plant species (*e*.*g*. [30–32]), while almost no research has focused on natural plant communities.

In this study we assessed the significance of chemical interference by an allelopathic plant in a natural semiarid plant community. Specifically, we conducted a 2-year field experiment to (1) determine the impacts of the allelopathic shrub *Artemisia herba-alba* Asso. (desert wormwood) [33,34] on plant growth and vitality, seed germination and seedling survival of neighboring species with and without the presence of chemical interference by the incorporation of AC, and to (2) assess the net interaction outcome for *A*. *herba-alba* in comparison with that for the similar but non-allelopathic shrub *Salsola vermiculata* L. (Mediterranean saltwort). *Artemisia herba-alba* and *S*. *vermiculata* have similar physiognomy and functional traits. Both are long-lived shrubs, having a moderately dense canopy, that provide a similar amelioration in resources availability (*e*.*g*. water and nutrients) and abiotic conditions (*e*.*g*. temperature) compared to bare soil areas [35]. We hypothesized that (i) chemical interference by *A*. *herba-alba* is an important process under field conditions. Specifically, we expected that plant growth, plant vitality, seed germination and seedling survival of neighboring species beneath the canopy of *A*. *herba-alba* would be enhanced through the addition of AC. Furthermore, we hypothesized that (ii) the allelopathic activity of *A*. *herba-alba* limits potential facilitative effects. In particular, we expected that the net interaction outcome for *S*. *vermiculata* would be positive, while that for *A*. *herba-alba* would be negative, even under very harsh abiotic conditions.

## Material and methods

### Study area

The study was conducted in the middle Ebro Valley (NE Spain), in the “El Planerón de Belchite” ornithological reserve (41°22'09''N, 00°37'50''W). This area is characterized by a semiarid Mediterranean climate, and has an average annual rainfall of 310 mm and average annual temperature of 15.4°C. Rainfall occurs mostly in spring and autumn, whereas winter and summer are usually dry (Panel A in S1 Fig). In the study area the summers are particularly warm (Panel B in S1 Fig), with the mean maximum summer temperatures exceeding 30°C, and the absolute temperature peaks at over 40°C (data obtained from the digital climatic atlas of Aragón; http://anciles.aragon.es/AtlasClimatico/). Hence, the vegetation is periodically subjected to severe water stress. Moreover, the soils have a very high clay content, which results in poor water infiltration and exacerbates water deficit. The main human activity in the area is traditional agro-pastoral land use [36]. The landscape consists of a mosaic of dry cereal crops and non-cultivated lands. The steppe plant community on non-cultivated lands is composed of small shrubs (*e*.*g*. *A*. *herba-alba*, *S*. *vermiculata* and *Suaeda vera* J.F.G.mel.), perennial grasses (*e*.*g*. *Dactylis glomerata* L. subsp. *hispanica* (Roth) Nyman, *Lygeum spartum* L. and *Stipa parviflora* Desf.) and several ephemeral herbs. The vegetation is clumped in patches within a matrix of bare soil. The area is freely accessible and approval to carry out the field work was obtained from the local SEO/BirdLife authorities.

### Experimental design

In early February 2014, 15 juvenile plants (< 1 year old) of *S*. *vermiculata*, *L*. *spartum* and *A*. *herba-alba* (n = 45), which are the three co-dominant species in the community, were transplanted in three different microsites: beneath the canopy of *A*. *herba-alba* adults, beneath adult individuals of *S*. *vermiculata* and in bare soil areas (> 50 cm to the nearest vegetation patch). Specifically, one juvenile was transplanted per *A*. *herba-alba* adult, *S*. *vermiculata* adult or bare soil (n = 135), maintaining the commercial soil where they germinated and grew in the nursery and avoiding southern exposure. In addition, 15 juveniles of *L*. *spartum* and *A*. *herba-alba* (n = 30) were transplanted in *A*. *herba-alba* and *S*. *vermiculata* microsites (n = 60) with the addition of AC (Activated Charcoal powder; PanReac AppliChem; Cu < 50 ppm, Fe < 500 ppm, Ni < 50 ppm, Pb < 50 ppm) to the soil at a concentration of 2% (*i*.*e*. 2 g of AC per 100 g of soil). The AC was added to the soil removed to form the planting hole and was thoroughly hand-mixed with that soil in a bucket before the soil was returned to the hole. *Salsola vermiculata* could not be transplanted in those two microsites with the addition of AC because not enough juveniles were available. Adult individuals of *A*. *herba-alba* and *S*. *vermiculata*, and bare soil areas were randomly selected in 1.3 ha of the study area having homogeneous slope and soil type. All juveniles were watered once following planting. Wild herbivores severely damaged some juveniles of *A*. *herba-alba* within few days of planting. Those were replaced and a metallic mesh was installed to prevent further grazing. Height and diameter of juveniles were measured at the beginning of the experiment and at the end of spring in three consecutive years; June 2014, June 2015 and June 2016. Then, the volume was calculated to estimate plant size. The volume of *S*. *vermiculata* and *A*. *herba-alba* juveniles was approximated to an inverted cone, while the volume of *L*. *spartum* juveniles was approximated to a cylinder. In addition, the vitality of juveniles was measured as an indicator of the general plant response to local stress conditions [37]. We defined vitality as the percentage of green aerial parts (0 for a dead juvenile) and was visually estimated in two contrasting drought conditions at the end of the experiment (at the end of spring, June 2016, and at the end of summer, October 2016). While spring 2016 was relatively rainy, drought stress was extremely high in the summer 2016 because there was almost no rainfall (Panel A in S1 Fig). The estimation of plant vitality was always performed by the same person, who had been trained by repeatedly assessing the vitality of control plants [37]. Juvenile plants of the three target species were supplied by a local garden center.

In December 2014, seeds of *S*. *vermiculata*, *L*. *spartum* and *A*. *herba-alba* were sown in *A*. *herba-alba*, *S*. *vermiculata* and bare soil microsites. Specifically, three seeds of *S*. *vermiculata*, two seeds of *L*. *spartum* or two seeds of A. *herba-alba* were sown beneath the same *A*. *herba-alba* and *S*. *vermiculata* adults, and bare soil areas where juveniles of these three target species had been transplanted (n = 15 individuals x 3 microsites x 2 seeds = 90 for *L*. *spartum* and *A*. *herba-alba*, and 15 individuals x 3 microsites x 3 seeds = 135 for *S*. *vermiculata*). In addition, seeds of target species were sown in *A*. *herba-alba* and *S*. *vermiculata* microsites with the addition of AC (n = 15 individuals x 2 microsites x 2 seeds = 60 for *L*. *spartum* and *A*. *herba-alba*, and 15 individuals x 2 microsites x 3 seeds = 90 for *S*. *vermiculata*). A higher number of *S*. *vermiculata* seeds was used because of the low establishment rate previously observed in a germination test. For the AC treatment, seeds were sown after thoroughly mixing 2 g of AC into 100 g of soil. In the control (*i*.*e*. without the addition of AC), seeds were sown following mixing of the same soil volume by hand. Seeds were sown at 1 cm depth, next to the transplanted juveniles, avoiding southern exposure. The position of each seed was marked by two nails to clearly identify the emerged seedling and distinguish it from other naturally emerging seedlings. Seed germination and the survival of emerged seedlings were recorded approximately every three months for 18 months (from December 2014 to June 2016 and from March 2015 to October 2016 respectively). All seeds were collected in the field from natural populations in the study area.

To control for potential side effects of AC, soil samples were collected in late October 2016 from 5 random juveniles of *L*. *spartum* and *A*. *herba-alba* transplanted beneath *A*. *herba-alba* adults with (n = 10) and without the addition of AC (n = 10) and beneath *S*. *vermiculata* adults with (n = 10) and without AC (n = 10). Organic carbon (C), total nitrogen (N), the C:N ratio, available phosphorous (P) and pH were analyzed for all soil samples. Soil samples were dried and sieved through a 2-mm mesh sieve prior to analysis. Organic C content was measured using chromatic acid digestion [38]. Total N and the C:N ratio were measured using a Vario MAX CN elemental analyzer. Available P was measured based on the absorbance at 430 nm (UNICAM 8625 UV/Vis Spectrometer) of samples extracted with Bray n°1 reagent [39]. Soil pH in water following a dilution 1:2.5 was measured using a pH meter CRISON micropH 2001.

### Net interaction outcome

The net interaction outcome for *A*. *herba-alba* and *S*. *vermiculata* microsites was estimated by computing the Relative Interaction Index (RII) [40] as:

$${RII}_{m,t}=\frac{V_{shrub}-V_{bare\;soil}}{V_{shrub}+V_{bare\;soil}},$$

where *V*_*shrub*_ is the value for the measured variables (plant size, plant vitality, seed germination or seedling survival) for each target species (*t* = *S*. *vermiculata*, *L*. *spartum* or *A*. *herba-alba*) in each shrub microsite (*m* = *A*. *herba-alba* or *S*. *vermiculata*) and *V*_*bare soil*_ is the value for these variables for the target species in the bare soil areas. The final size of juveniles rather than growth was used to calculate RII, because a negative change in growth was observed for some juvenile plants and negative values cannot be used to calculate RII properly [41]. Nevertheless, the initial size of the transplanted juveniles of target species did not differ significantly among microsites (*p* > 0.05 for the three target species; S1 Table). The RII for plant vitality was estimated under two drought stress conditions (*i*.*e*. spring and summer 2016). Measuring the size of juveniles after the summer of 2016 was not meaningful because they do not grow during this season. For seed germination and seedling survival, a global RII was calculated per target species and shrub microsite using the values at the end of the experiment.

The RII values range between 1 and -1. Positive RII values indicate that the measured variables are higher in the shrub microsite than in the bare soil, and thus, net facilitative effects can be inferred. Negative RII values indicate that the variables are lower in the shrub microsite than in the bare soil, indicating net interference. RII values of approximately zero indicate that the measured variables are similar in the shrub microsite and bare soil areas.

### Statistical analysis

Differences in plant growth and vitality of juveniles between treatments (with and without the addition of AC) and shrub microsites (*A*. *herba-alba* and *S*. *vermiculata*) were analyzed, for each target species, using linear mixed models (LMMs) for repeated measures. Treatment, microsite and year of measurement were set as fixed factors, while the identity of each juvenile was included as a random factor. Only juveniles that survived to the end of the experiment were included in the assessment of plant growth. Data on vitality of juveniles was arcsine transformed to meet normality assumptions.

Seed germination and survival of emerged seedlings were modeled, for each target species, as time functions using the Kaplan-Meier method. The significance of differences in curves shape between treatments (with and without AC) and microsites (*A*. *herba-alba* and *S*. *vermiculata*) was tested using Cox regressions [42].

Differences in soil organic C, total N, the C:N ratio, available P and pH between treatments (with and without AC) and shrub microsites (*A*. *herba-alba* and *S*. *vermiculata*) were analyzed using two-way ANOVAs. The organic C and total N data was arcsine transformed to meet normality assumptions.

The presence of facilitative, neutral or negative interactions based on the RII values for the size and vitality of juveniles was analyzed by performing Student t-tests against a constant value (zero) [4]. Furthermore, significant differences in RII values between shrub microsites were analyzed by pooling all species together and for each target species separately, using two-way and one-way ANOVA respectively. Because only one RII value was calculated for seed germination and the survival of emerged seedlings for each combination of target species and shrub microsite, there was a lack of variability to statistically test differences between shrub microsites. To overcome this problem, the germination and survival were simulated 100 times for each target species and microsite using bootstrapping [43]. Specifically, in each simulation, the germination was calculated by resampling the 15 individuals per shrub and bare soil microsites with replacement to obtain realistic values of germination in the microsites. The same resampling was performed to simulate survival, but only those shrub individuals and bare soil areas that had at least one germinated seed were considered (if germination was zero, it was not possible to calculate the survival). The RII was subsequently computed for each simulation. The RII values between shrub microsites were compared by calculating the z-score for the differences between microsites and simulations [43].

Statistical analyses were performed in R [44]. LMMs were performed using the *lme* function in the *nlme* package [45]. Germination and survival curves were performed using the *survfit* function in the *survival* package [46].

## Results

### Effects of chemical interference on neighboring plant species

**Fig 1:**
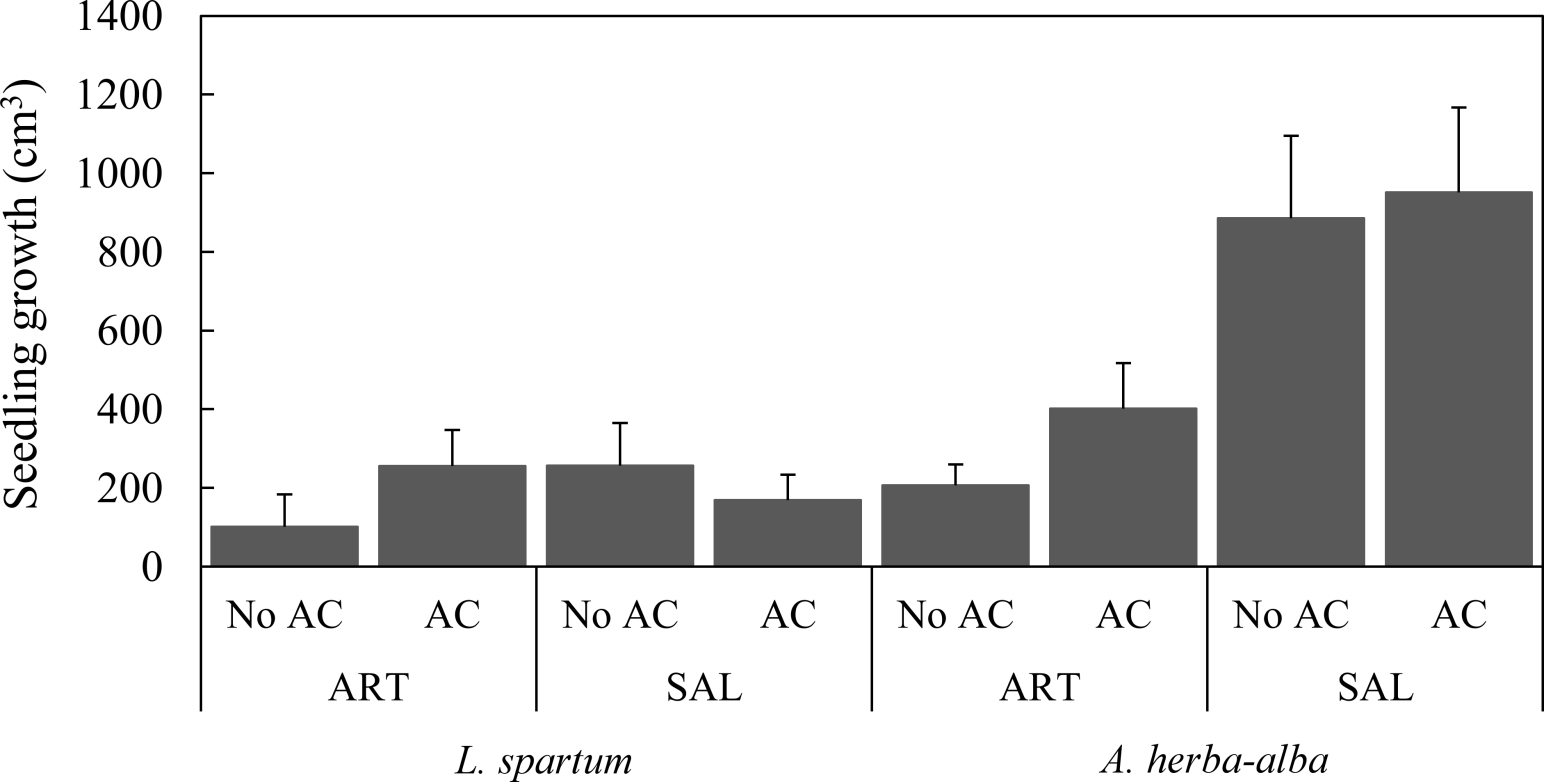
Annual growth (mean ± SE) of target species juveniles by treatment and shrub microsite. ART, *A*. *herba-alba*; SAL, *S*. *vermiculata*.

The growth of *L*. *spartum* and *A*. *herba-alba* juveniles transplanted with the addition of AC did not differ significantly from those transplanted without AC (Table 1; Fig 1). In addition, growth of *L*. *spartum* juveniles did not differ between microsites (Table 1; Fig 1). However, the growth of *A*. *herba-alba* juveniles transplanted in *S*. *vermiculata* microsite was significantly higher than those transplanted in *A*. *herba-alba* microsite (Table 1; Fig 1). On the other hand, growth of juveniles differed significantly among years of measurement (Table 1). Specifically, growth of *L*. *spartum* juveniles was significantly less in 2015 than in 2014 and 2016, while growth of *A*. *herba-alba* juveniles was significantly higher in 2016 than in 2014 (S2 Fig).

**Table 1:** Result of LMM analyses for the effect of treatment, microsite and time of measurement on the growth and vitality of target species.

	*L*. *spartum*				*A*. *herba-alba*			
	Df	Sum Sq	F value	*p* (>F)	Df	Sum Sq	F value	*p* (>F)
Plant growth								
Treatment	1	1.86 x 10^4^	0.087	0.77	1	6.20 x 10^5^	0.533	0.47
Microsite	1	2.90 x 10^4^	0.136	0.74	1	1.66 x 10^7^	14.281	**< 0.001**
Year	2	5.15 x 10^6^	12.077	**< 0.001**	2	8.04 x 10^6^	3.453	**0.04**
Treatment:Microsite	1	4.76 x 10^5^	2.231	0.14	1	1.87 x 10^5^	0.161	0.69
Treatment:Year	2	5.22 x 10^5^	1.224	0.30	2	3.51 x 10^6^	1.506	0.23
Microsite:Year	2	2.81 x 10^5^	0.659	0.52	2	3.62 x 10^6^	1.555	0.22
Plant vitality								
Treatment	1	0.086	0.771	0.38	1	0.275	2.372	0.13
Microsite	1	0.040	0.358	0.55	1	0.213	1.839	0.18
Season	1	10.314	92.163	**< 0.001**	1	7.29	62.946	**< 0.001**
Treatment:Microsite	1	0.053	0.476	0.49	1	0.514	4.437	**0.04**
Treatment:Season	1	0.019	0.168	0.68	1	0.058	0.498	0.48
Microsite:Season	1	0.730	6.524	**0.01**	1	0.270	2.331	0.13

Significant effects (*p* < 0.05) are highlighted in bold.

**Fig 2:**
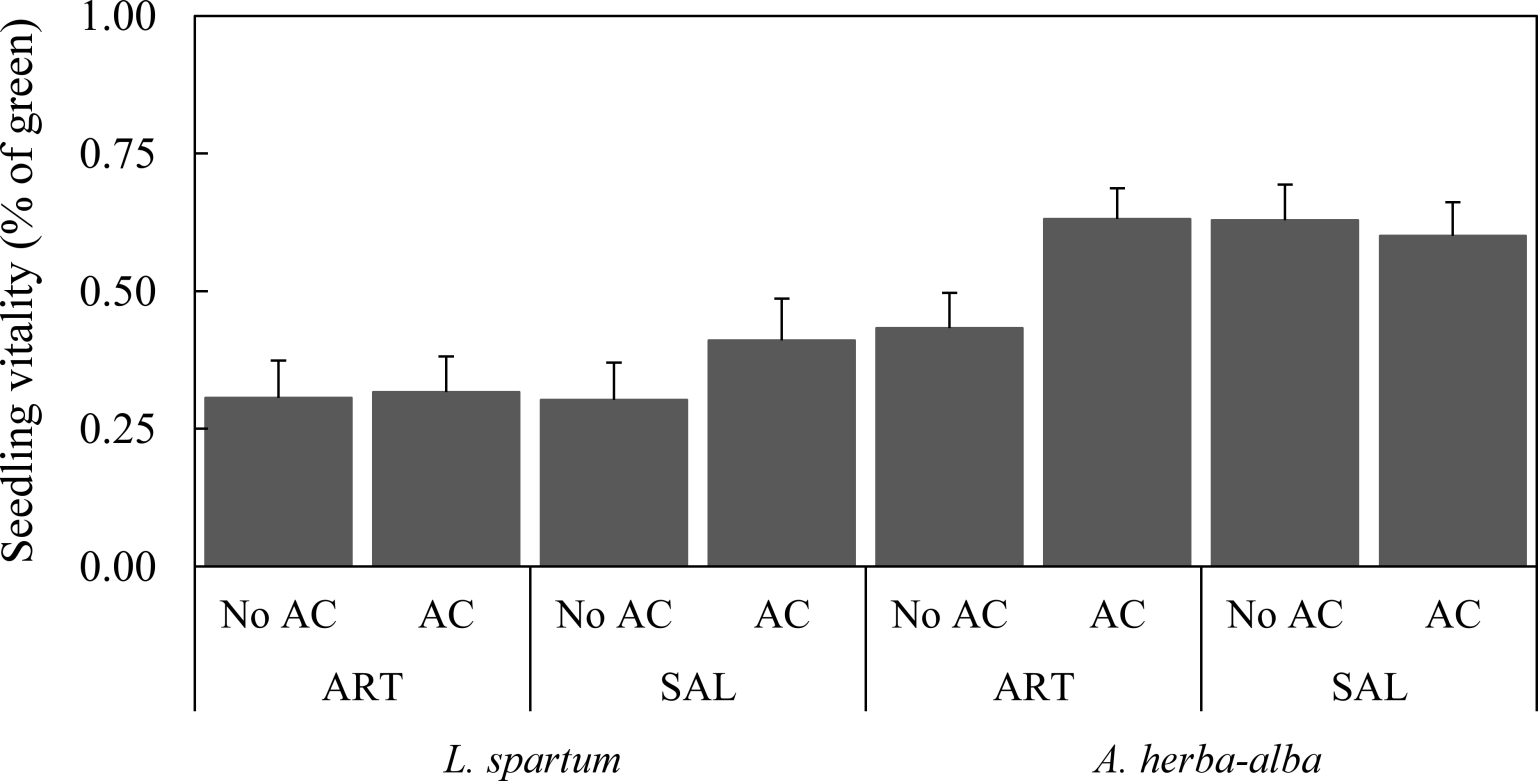
Vitality (mean % of green parts ± SE) of target species juveniles by treatment and shrub microsite. ART, *A*. *herba-alba*; SAL, *S*. *vermiculata*.

Overall, vitality (% of green plant parts) of *L*. *spartum* and *A*. *herba-alba* juveniles did not differ significantly between treatments or microsites (Table 1; Fig 2). However, there was a significant interaction between treatment and microsite regarding *A*. *herba-alba* juveniles (Table 1). While vitality of *A*. *herba-alba* juveniles transplanted in *A*. *herba-alba* microsite amended with AC was higher than those in the absence of AC, vitality of *A*. *herba-alba* juveniles transplanted in *S*. *vermiculata* microsite did not differ between treatments (Fig 2). On the other hand, vitality of juveniles was significantly lower under more severe drought stress conditions (*i*.*e*. after summer; Table 1; S3 Fig).

**Fig 3:**
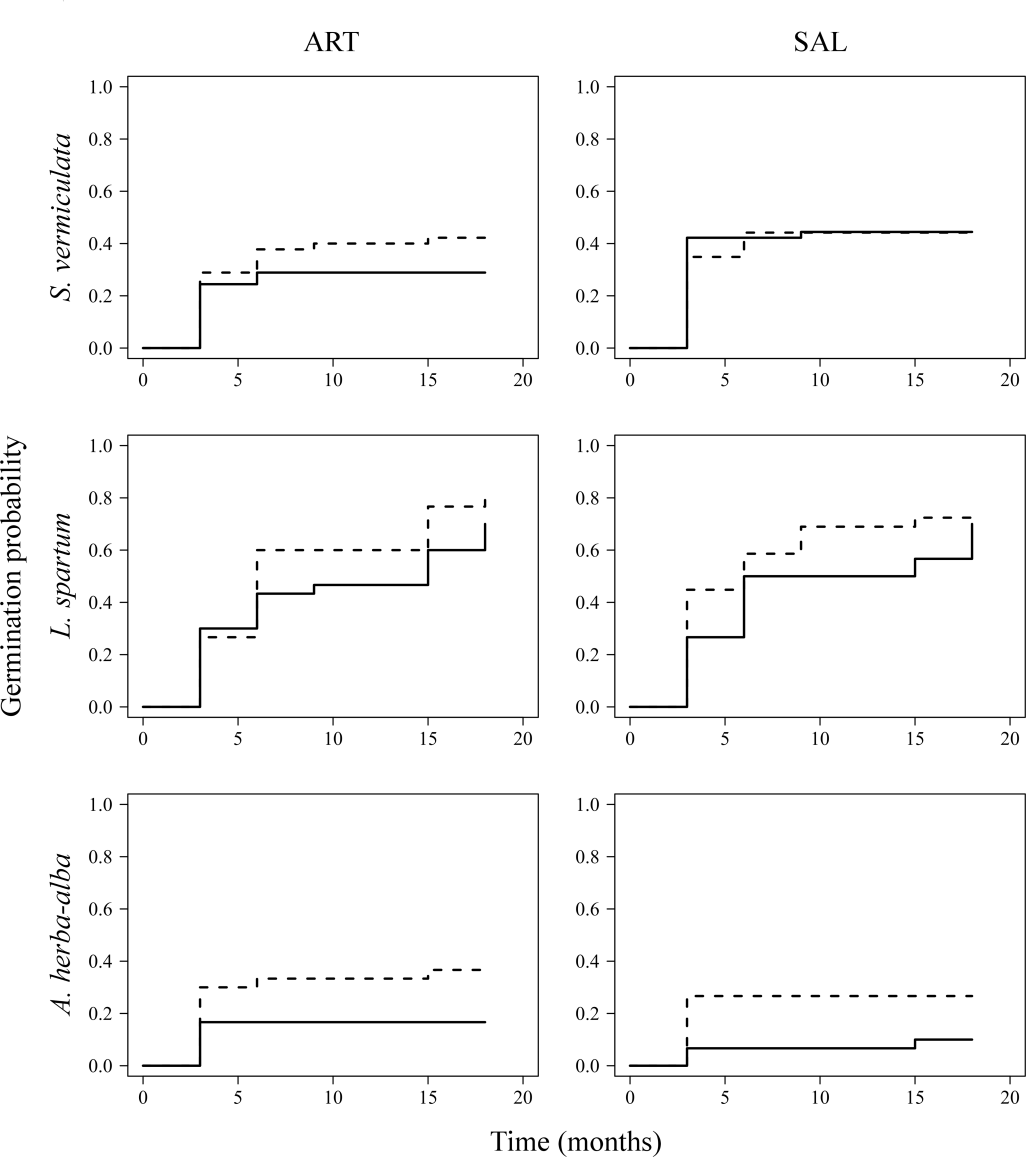
Seed germination (Kaplan-Meier curves) of target species by treatment and shrub microsite. Dashed and solid lines represent germination of seeds sowed with and without the addition of AC respectively. ART, *A*. *herba-alba*; SAL, *S*. *vermiculata*.

**Fig 4:**
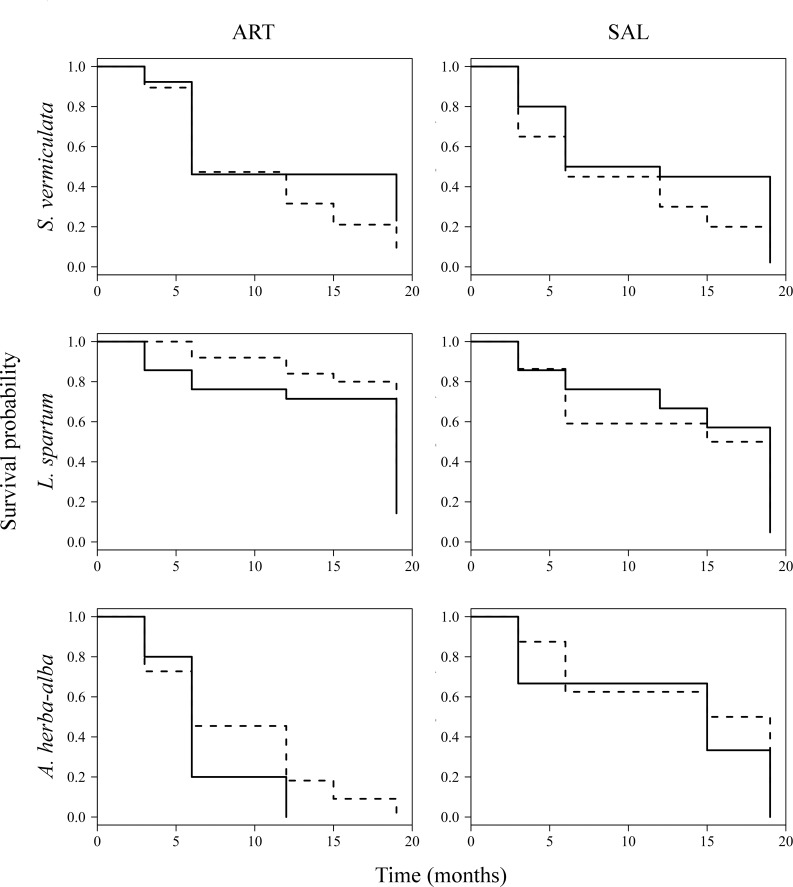
Seedling survival (Kaplan-Meier curves) of target species by treatment and shrub microsite. Dashed and solid lines represent germination of seeds sowed with and without the addition of AC respectively. ART, *A*. *herba-alba*; SAL, *S*. *vermiculata*.

Significant effects of AC were not observed on germination of *S*. *vermiculata* and *L*. *spartum* seeds (Table 2; Fig 3). However, germination of *A*. *herba-alba* seeds sowed with the addition of AC was significantly higher than those sowed without AC, regardless of the microsite (Table 2; Fig 3). In addition, significant differences in seed germination between microsites were not found for any of the three target species (Table 2; Fig 3). On the other hand, seedling survival of target species did not differ significantly between treatments (Table 2; Fig 4). However, survival of *L*. *spartum* and *A*. *herba-alba* seedlings differed significantly between microsites. Specifically, survival of *L*. *spartum* seedlings emerged in *A*. *herba-alba* microsite was significantly higher than those emerged in *S*. *vermiculata* microsite (Table 2; Fig 4), while survival of *A*. *herba-alba* seedlings emerged in *A*. *herba-alba* microsite was significantly lower than those emerged in *S*. *vermiculata* microsite (Table 2; Fig 4).

**Table 2:** Result of Cox regressions for the effect of treatment and microsite on the germination and survival of target species.

	*S*. *vermiculata*				*L*. *spartum*				*A*. *herba-alba*			
	loglik	χ^2^	df	*p*	loglik	χ^2^	df	*p*	loglik	χ^2^	df	*p*
Seed germination												
Treatment	-355.97	0.651	1	0.42	-377.24	2.259	1	0.13	-123.16	5.875	1	**0.02**
Microsite	-355.26	2.060	1	0.15	-377.24	5 x 10^−4^	1	0.98	-122.59	1.123	1	0.29
Treatment:Microsite	-354.55	0.789	1	0.37	-377.24	3 x 10^−4^	1	0.99	-122.56	0.068	1	0.79
Seedling survival												
Treatment	-233.10	2.178	1	0.14	-281.58	2.801	1	0.09	-64.28	0.554	1	0.46
Microsite	-233.54	1.291	1	0.26	-279.42	4.313	1	**0.04**	-61.78	5.005	1	**0.03**
Treatment:Microsite	-232.20	0.112	1	0.74	-278.87	1.102	1	0.29	-61.73	0.092	1	0.76

Significance of the change in deviance was tested by comparing models with and without the effect of each term, assuming a χ^2^ distribution. Significant effects (*p* < 0.05) are highlighted in bold.

Organic C content and the C:N ratio were significantly higher in soils amended with AC than in soils without the addition of AC (Table 3). Furthermore, pH was significantly lower in soils having added AC than in soils without added AC (Table 3). Total N and available P content did not differ significantly between treatments (Table 3). On the other hand, only available P content in soil differed significantly between microsites, being higher in *S*. *vermiculata* microsite than it was in *A*. *herba-alba* microsite (Table 3).

**Table 3:** Soil chemical characteristics (mean ± SE) and result of ANOVA analyses for the effect of treatment and microsite on those characteristics.

	ART		SAL	
	No AC	AC	No AC	AC
Organic C (%)	1.13 ± 0.14	1.99 ± 0.14	1.57 ± 0.37	2.03 ± 0.19
Total N (%)	0.103 ± 0.012	0.113 ± 0.020	0.174 ± 0.042	0.134 ± 0.012
C:N	11.26 ± 0.80	18.85 ± 2.21	9.74 ± 1.10	15.44 ± 0.95
Available P (mg/kg)	46.09 ± 6.64	61.76 ± 8.28	85.97 ± 5.63	88.15 ± 7.37
pH	7.92 ± 0.04	7.67 ± 0.02	7.94 ± 0.03	7.71 ± 0.02
	Df	Sum Sq	F value	p (>F)
Organic C (%)				
Treatment	1	0.009	11.189	**< 0.01**
Microsite	1	5.85 x 10^−4^	0.759	0.39
Treatment:Microsite	1	4.72 x 10^−4^	0.612	0.44
Residuals	36	0.028		
Total N (%)				
Treatment	1	4 x 10^−7^	0.004	0.95
Microsite	1	2.39 x 10^−4^	2.603	0.12
Treatment:Microsite	1	8.12 x 10^−5^	0.884	0.35
Residuals	36	0.003		
C:N				
Treatment	1	441.36	23.156	**< 0.001**
Microsite	1	60.77	3.188	0.08
Treatment:Microsite	1	8.94	0.469	0.50
Residuals	36	686.18		
Available P (mg/kg)				
Treatment	1	795.8	1.603	0.21
Microsite	1	1.098 x 10^4^	22.108	**< 0.001**
Treatment:Microsite	1	454.8	0.916	0.35
Residuals	36	1.788 x 10^4^		
pH				
Treatment	1	0.571	80.984	**< 0.001**
Microsite	1	0.006	0.886	0.35
Treatment:Microsite	1	0.001	0.172	0.68
Residuals	36	0.007		

ART, *A*. *herba-alba* microsite; SAL, *S*. *vermiculata* microsite; C, carbon; N, nitrogen; P, phosphorous. Significant effects (*p* < 0.05) are highlighted in bold.

### Net interaction outcome

**Fig 5:**
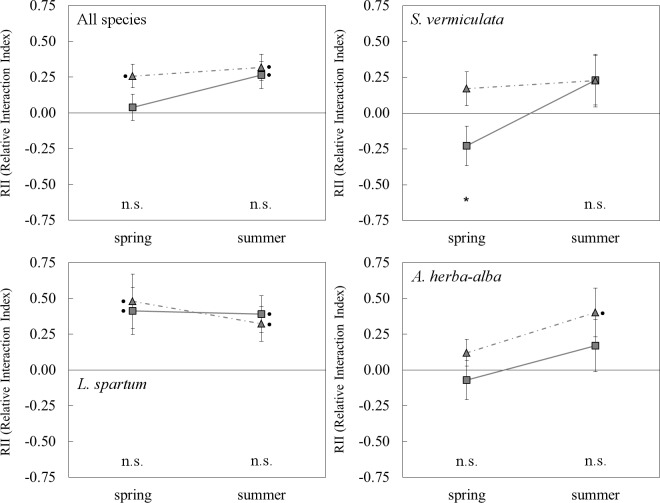
Net interaction outcome (RII; mean ± SE) of shrub microsites in spring and summer. Square and triangle symbols indicate RII values calculated for the vitality of juveniles in *A*. *herba-alba* and *S*. *vermiculata* microsites respectively. Black dots indicate RII values significantly different from zero (t-test, *p* < 0.05). n.s., not significant; *, *p* < 0.05.

The RII value for the size of juveniles indicated a neutral interaction outcome for *A*. *herba-alba* and *S*. *vermiculata* microsites (*t*-test, *p* > 0.05; Panel A in S4 Fig). Furthermore, non-significant differences were observed in the RII values between these two microsites (*p* > 0.05; Panel A in S4 Fig). On the other hand, the RII values for the vitality of juveniles indicated an overall neutral interaction outcome for the effect of *A*. *herba-alba* on target species, but a net positive outcome for the effect of *S*. *vermiculata* (Fig 5). The difference in the RII values between the two microsites was marginally significant (Table 4). Furthermore, the interaction outcome for the *A*. *herba-alba* microsite changed from neutral in spring to positive in the summer, when it was similar to the summer interaction outcome for the *S*. *vermiculata* microsite (Table 4; Fig 5). This general pattern varied slightly depending on the target species. Specifically, *A*. *herba-alba* and *S*. *vermiculata* had neutral net effects on *S*. *vermiculata* juveniles in both drought stress conditions (Fig 5), although in spring the RII value was significantly higher for the *S*. *vermiculata* microsite than for the *A*. *herba-alba* microsite (Table 4). Similarly, we found neutral effects of each shrub species on *A*. *herba-alba* juveniles in spring. However, the net interaction outcome for these juveniles became positive in the summer in the *S*. *vermiculata* microsite, while a neutral net effect was found in summer for *A*. *herba-alba* juveniles in the *A*. *herba-alba* microsite. Differences in RII values between the two microsites were non-significant (Table 4). On the other hand, the *A*. *herba-alba* and *S*. *vermiculata* microsites had net facilitative interactions on *L*. *spartum* juveniles. This positive interaction outcome remained constant under the more severe drought stress conditions (Table 4; Fig 5).

**Table 4:** Result of ANOVA analyses for the net interaction outcome of shrub microsites under the two drought stress conditions.

	Spring				Summer			
	Df	Sum Sq	F value	*p* (>F)	Df	Sum Sq	F value	*p* (>F)
All species								
Microsite	1	1.080	3.107	0.08	1	0.065	0.169	0.68
Species	2	4.044	6.552	**<0.01**	2	0.247	0.318	0.73
Residuals	88	30.588			88	33.667		
*S*. *vermiculata*								
Microsite	1	1.194	4.793	**0.04**	1	1 x 10^−4^	1 x 10^−4^	0.99
Residuals	28	6.977			28	13.408		
*L*. *spartum*								
Microsite	1	0.034	0.072	0.79	1	0.035	0.150	0.70
Residuals	28	13.290			28	6.588		
*A*. *herba-alba*								
Microsite	1	0.273	1.307	0.26	1	0.404	0.867	0.36
Residuals	28	5.846			28	13.050		

RII index calculated for the vitality of juveniles. Significant differences (*p* < 0.05) are highlighted in bold.

The RII values calculated for seed germination and seedling survival indicated a neutral interaction outcome in the *A*. *herba-alba* and *S*. *vermiculata* microsites (Panels B and C in S4 Fig). In addition, in no case did RII values calculated for these two variables differ significantly between *A*. *herba-alba* and *S*. *vermiculata* microsites (*p* > 0.05; Panels B and C in S4 Fig).

## Discussion

We conducted a 2-year field experiment to determine the significance of chemical interference by the allelopathic plant *A*. *herba-alba* in a semiarid plant community, by assessing its impacts on the growth, vitality, seed germination and seedling survival of neighboring plant species. We also investigated how allelopathy modulates the net interaction outcome for *A*. *herba-alba*. Previous research has shown that *A*. *herba-alba* exerts a negative influence on surrounding vegetation. In an observational study, Friedman *et al*. [33] found that germination of annuals plants was suppressed in the vicinity of *A*. *herba-alba*. In the same line, Pueyo *et al*. [35] found a generalized spatial pattern of segregation between adult individuals of *A*. *herba-alba* and juvenile individuals of perennial plant species, and also found that *A*. *herba-alba* had a relatively low density of other plants beneath its canopy. In agreement, Arroyo *et al*. [47] found less species in the local neighbor of *A*. *herba-alba* individuals than expected. In these studies the authors argued that the negative effects of *A*. *herba-alba* on vegetation might be a consequence, at least partially, of its allelopathic activity. This was based on a number of laboratory studies that demonstrated the phytotoxicity of the compounds released by this species [33,48–50]. However, these observational studies provided no direct evidence of allelopathic interference, as they lacked an adequate control to remove the inhibitory compounds (*e*.*g*. AC). In our experimental field study, we found that the chemical interference of *A*. *herba-alba* affected some neighboring plant species beneath its canopy (*i*.*e*. vitality), supporting previous conclusions.

The addition of AC to soil in studies of allelopathy has been used to reduce or prevent chemical interference, because it adsorbs the phytotoxic compounds released by allelopathic plants [22,51]. However, it is imperative to control for other potential effects of the use of AC which could confuse interpretation of the positive effects resulting from release from allelopathic interference [52]. In this sense, we found that germination of *A*. *herba-alba* seeds sowed in *A*. *herba-alba* microsite was enhanced by amending the soil with AC. This finding would agree with a previous study conducted under controlled conditions, which showed that volatile and water soluble chemicals released from *A*. *herba-alba* inhibited the germination of its own seeds [48]. However, we also found that germination of *A*. *herba-alba* seeds was stimulated in *S*. *vermiculata* microsite with the addition of AC, which suggests that the enhanced germination in *A*. *herba-alba* microsite amended with AC was not due to a reduction in its chemical interference, but some AC side effect. Actually, the addition of AC (2%) modified the soil pH, increased the organic C content and, accordingly, the C:N ratio. However, in these environments, it has been observed that germination is usually ubiquitous across microsites (*e*.*g*. shrub vs. open bare soil), even though the amount of nutrients (*e*.*g*. amount of P) varies [35,53,54]. In agreement, we found no differences in germination rate of target species between microsites. Therefore, it is unlikely that the enhancement of germination was due to changes in those soil parameters. Alternatively, the addition of AC to the soil can result in texture changes that would increase water retention capacity, because of the finer particle size [22]. Although in our study we did not evaluate this possibility, it seems the most plausible explanation.

On the other hand, we observed that seedling survival of target species emerged in *A*. *herba-alba* microsite without the addition of AC was not hampered in comparison with *A*. *herba-alba* microsite amended with AC. However, a previous laboratory study reported that the aqueous extract of *A*. *herba-alba* increased mortality of emerging *A*. *herba-alba* seedlings from the seedbank [34]. Similarly, we did not find any effect of AC treatment on the growth of juvenile plants, despite a previous laboratory study showing that both volatile and water soluble chemicals released by *A*. *herba-alba* enhanced early seedling growth [48]. Differences in the allelopathic effects of *A*. *herba-alba* between previous laboratory studies and our field experiment might be because of differences in the experimental conditions, including the soil microbial community, level of exposure to allelopathic compounds, the specific mixture or the relative concentrations of compounds acting jointly under natural conditions. For instance, it is known that in the field, associated soil microorganisms can metabolize chemical compounds and diminish their allelopathic effects [55,56]. This emphasizes that laboratory studies might overestimate the significance of allelopathy in nature, as discussed in previous studies [57–60]. Consequently, results obtained under controlled conditions should be interpreted carefully. However, it should be noted that growth and survival of *A*. *herba-alba* (particularly sensitive to its own allelopathic compounds, [34,48]) was higher in *S*. *vermiculata* microsite than they were in *A*. *herba-alba* microsite, while survival of *L*. *spartum* seedlings (rather tolerant to *A*. *herba-alba* chemicals, [48]) was higher beneath *A*. *herba-alba* than in *S*. *vermiculata* microsite. These findings would suggest somehow that AC might not be reducing chemical interference enough for these two aspects of plant performance.

Our results showed that the main impact of chemical interference by *A*. *herba-alba* was a reduction in plant vitality of some neighboring plant species. In accordance, there were no differences in the RII values calculated for plant growth, seed germination and seedling survival between *A*. *herba-alba* and *S*. *vermiculata*. Only the RII values for plant vitality indicated different effects of these two species in plant biotic interactions. Specifically, our results indicated a neutral net interaction outcome for *A*. *herba-alba* in this semiarid plant community. This finding is consistent with that of Arroyo *et al*. [47], who found that *A*. *herba-alba* had a neutral net effect on plant diversity in the same semiarid community. On the other hand, the non-allelopathic shrub *S*. *vermiculata* had a net positive interaction outcome on target species, in agreement with previous studies showing that *S*. *vermiculata* is an effective nurse plant [35,47]. In other words, while plant vitality in *S*. *vermiculata* microsite was greater than it was in the bare soil, plant vitality in *A*. *herba-alba* microsite not. Having a similar species with no phytotoxicity has been considered as an adequate control to compare the effects of allelopathic species, particularly in field studies [57]. In the study area, both *A*. *herba-alba* and *S*. *vermiculata* provide a similar amelioration of microclimatic conditions beneath their canopies with respect to bare soil areas. In particular, it has been reported that soil water content and infiltration rate are substantially higher beneath the canopy of individuals of these two species than in bare soil, while solar radiation and maximum temperature are lower in comparison with bare soil areas (see [35] for further details). Therefore, differences in the net interaction outcome between *A*. *herba-alba* and *S*. *vermiculata* can be attributed to the allelopathic activity of *A*. *herba-alba* rather than differences in microclimatic conditions. Hence, it seems that chemical interference can limit somewhat the potential facilitative effects of enhanced microclimatic conditions in *A*. *herba-alba* microsite.

During summer 2016 the drought stress conditions became extreme, as almost no rainfall occurred in the study area between June and September 2016. It has been reported that a shift from neutral (or even positive) to negative effects occurs in the interaction outcome for allelopathic species under very harsh drought stress conditions [61], in accordance with predictions of the refined stress gradient hypothesis (SGH) [14]. However, we found that the outcome of interactions between *A*. *herba-alba* and neighboring species shifted from neutral to positive. Soil water scarcity may have limited the diffusion of water-borne allelopathic compounds to neighboring plants [62]. Consequently, allelopathy did not prevent a shift to a positive outcome under the more severe drought conditions. Thus, facilitative interactions predominated over interference (*i*.*e*. competition + allelopathy) of the allelopathic plant under very harsh abiotic stress, regardless of whether production or the phytotoxicity of allelopathic compounds increased under those conditions [15,16].

Our results showed that the balance between facilitative interactions and interference of *A*. *herba-alba* was species-specific. We found a neutral net effect on *S*. *vermiculata* and *A*. *herba-alba* juveniles, but, interestingly, net facilitative interactions were found for *L*. *spartum* juveniles in *A*. *herba-alba* microsite, regardless of drought severity. Indeed, other perennial grasses in addition to *L*. *spartum* may tolerate the allelopathic compounds of *A*. *herba-alba* [34,35,47]. Perhaps species of this group have become adapted to the chemicals of *A*. *herba-alba* as a consequence of a long associational history [18,63]. Thus, in this semiarid plant community *A*. *herba-alba* may act as a nurse plant facilitating the establishment of perennial grasses, which once established may replace their own nurse plants [64]. This highlights the potential facilitative effects that an allelopathic species can have in a semiarid plant community, despite chemical interference effects on some species.

In conclusion, this study of allelopathy constitutes one of the few examples performed in natural plant communities (but see [57,65,66]) and provides novel evidence for the significance of the chemical interference by an allelopathic shrub in a semiarid plant community. Our findings indicate that under natural conditions chemical interference can result in a reduction of the vitality of some neighboring plant species (*e*.*g*. *A*. *herba-alba*), supporting our first hypothesis. In addition, they suggest that allelopathy can limit potential facilitative effects of enhanced microclimatic conditions beneath allelopathic shrubs in semiarid environments, in agreement with our second hypothesis, although, tolerant species (*e*.*g*. *L*. *spartum*) may still being facilitated. This highlights the importance of a multispecific perspective in studies of allelopathy. Our results also show that net facilitative interactions should be expected for allelopathic shrubs under very harsh drought stress conditions. Our study links the observed effects in the field of the allelopathic shrub *A*. *herba-alba* on vegetation pattern, with laboratory studies that have demonstrated the phytotoxic properties of this plant. Although methods to test chemical interference may result challenging, further field studies are required to better understand the impacts of chemical interference by allelopathic plant species in natural plant communities.

## Supporting information

S1 Fig Climatic profile of the study area. (A) Monthly rainfall (solid lines) in the study area during the period 2014–2016. The shaded area represents the average rainfall over 30 years (1970–2000). (B) Average monthly temperature (solid lines) in the study area during the period 2014–2016. The dashed line represents the average temperature over 30 years (1970–2000). Data for the period 2014–2016 were obtained from the nearest meteorological station (Belchite station; http://eportal.magrama.gob.es/websiar/SeleccionParametrosMap.aspx?dst=1). Data for the period 1970–2000 were obtained from the digital climatic atlas of Aragón (http://anciles.aragon.es/AtlasClimatico/).
(TIFF)

S2 Fig Annual growth (mean ± SE) of target species juveniles by year. Different letters indicate significant differences among years (Tukey's HSD test; *p* < 0.05).
(TIF)

S3 Fig Vitality (mean % of green parts ± SE) of target species juveniles by season and shrub microsite. ART, *A*. *herba-alba*; SAL, *S*. *vermiculata*.
(TIF)

S4 Fig Net interaction outcome of shrub microsites. Relative interaction index (RII) calculated for (A) the size (mean ± SE), (B) germination and (C) survival of the target species in *A*. *herba-alba* (square symbols) and *S*. *vermiculata* (triangle symbols) microsites. Bars in B) and C) indicate the 95% confidence interval obtained using the bootstraping (see material and methods section for further details). n.s., not significant.
(TIFF)

S1 Table Initial size (cm^3^, mean ± SE) of target species juveniles transplanted by microsite. ART, *A*. *herba-alba*; SAL, *S*. *vermiculata*; BS, bare soil. Significant differences among microsites (ANOVA, *p* < 0.05) are highlighted in bold.
(PDF)

S1 Appendix Database. This appendix contains juvenile size and vitality data, seed germination and seedling survival data, and soil chemical data used.
(XLSX)
