# Supplementary figures and images for: Evidence for chemical interference effect of an allelopathic plant on neighboring plant species: A field study
Source: PLoS One. 2018 Feb 23;13(2):e0193421. doi: 10.1371/journal.pone.0193421 (PMC5825076; doi:10.1371/journal.pone.0193421)

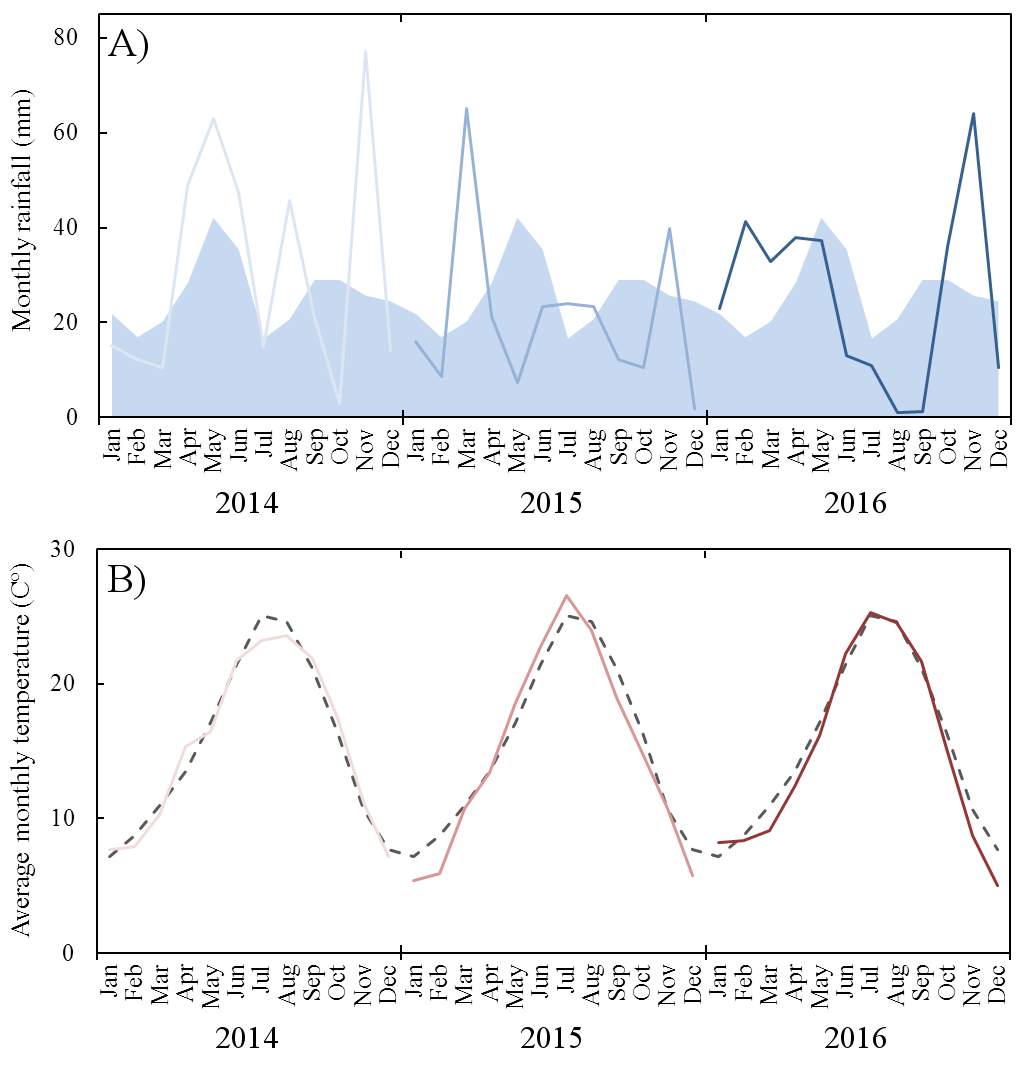

Supplement: S1 Fig — (A) Monthly rainfall (solid lines) in the study area during the period 2014–2016. The shaded area represents the average rainfall over 30 years (1970–2000). (B) Average monthly temperature (solid lines) in the study area during the period 2014–2016. The dashed line represents the average temperature over 30 years (1970–2000). Data for the period 2014–2016 were obtained from the nearest meteorological station (Belchite station; http://eportal.magrama.gob.es/websiar/SeleccionParametrosMap.aspx?dst=1). Data for the period 1970–2000 were obtained from the digital climatic atlas of Aragón (http://anciles.aragon.es/AtlasClimatico/). (TIFF) [file pone.0193421.s001.tiff]

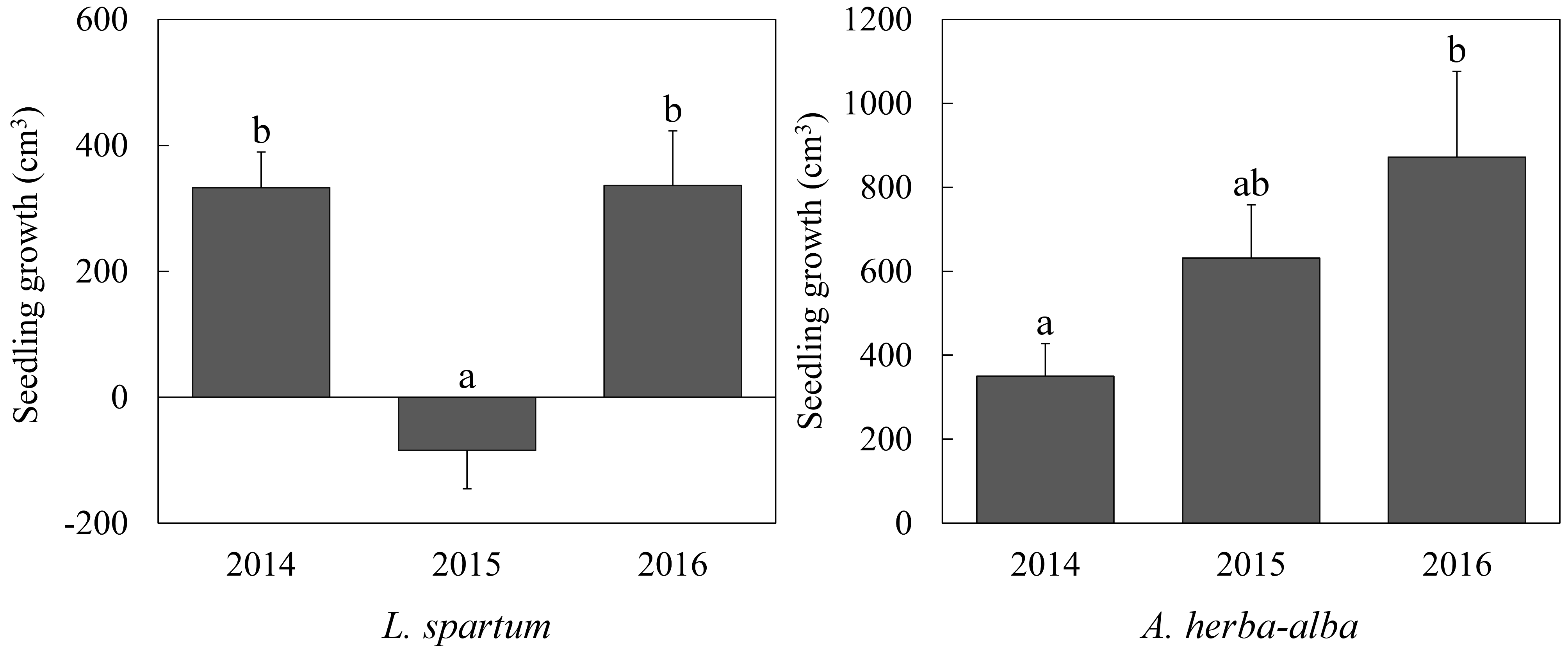

Supplement: S2 Fig — Different letters indicate significant differences among years (Tukey's HSD test; p < 0.05). (TIF) [file pone.0193421.s002.tif]

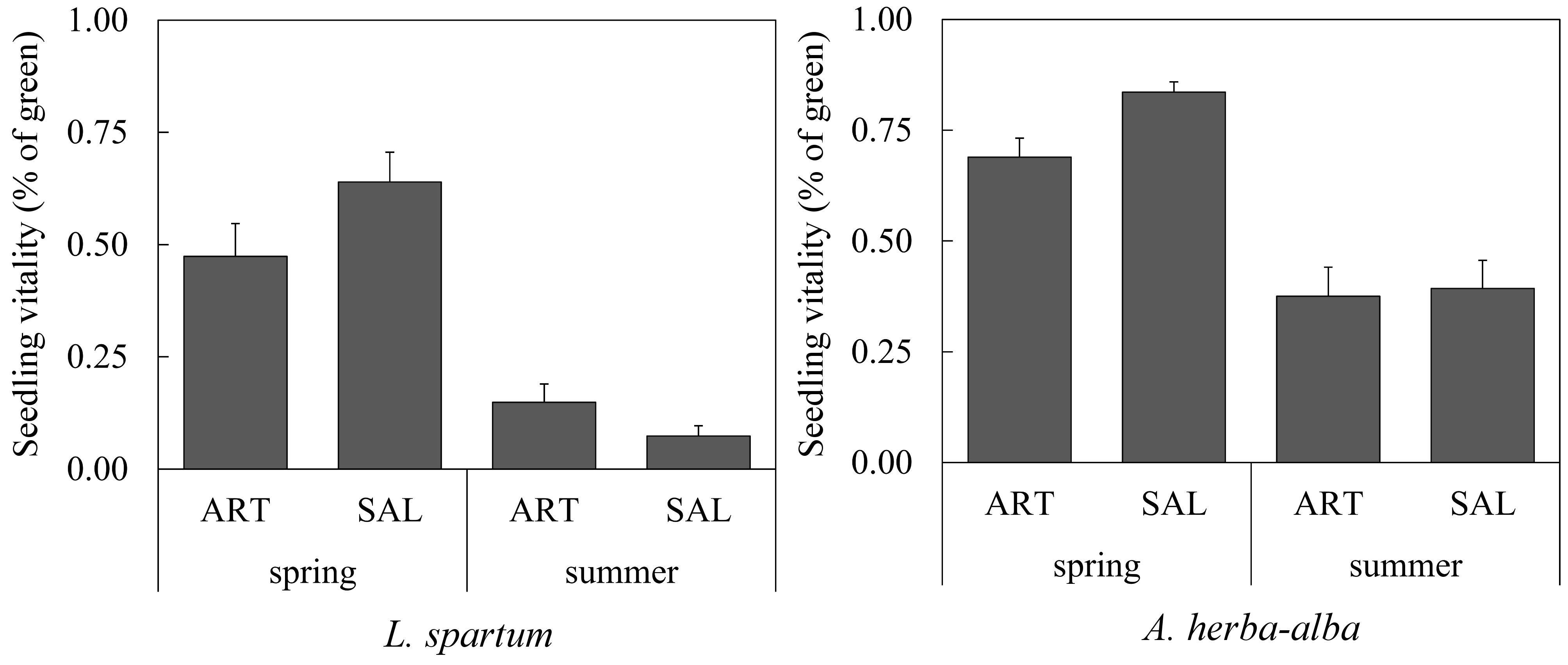

Supplement: S3 Fig — ART, A. herba-alba; SAL, S. vermiculata. (TIF) [file pone.0193421.s003.tif]

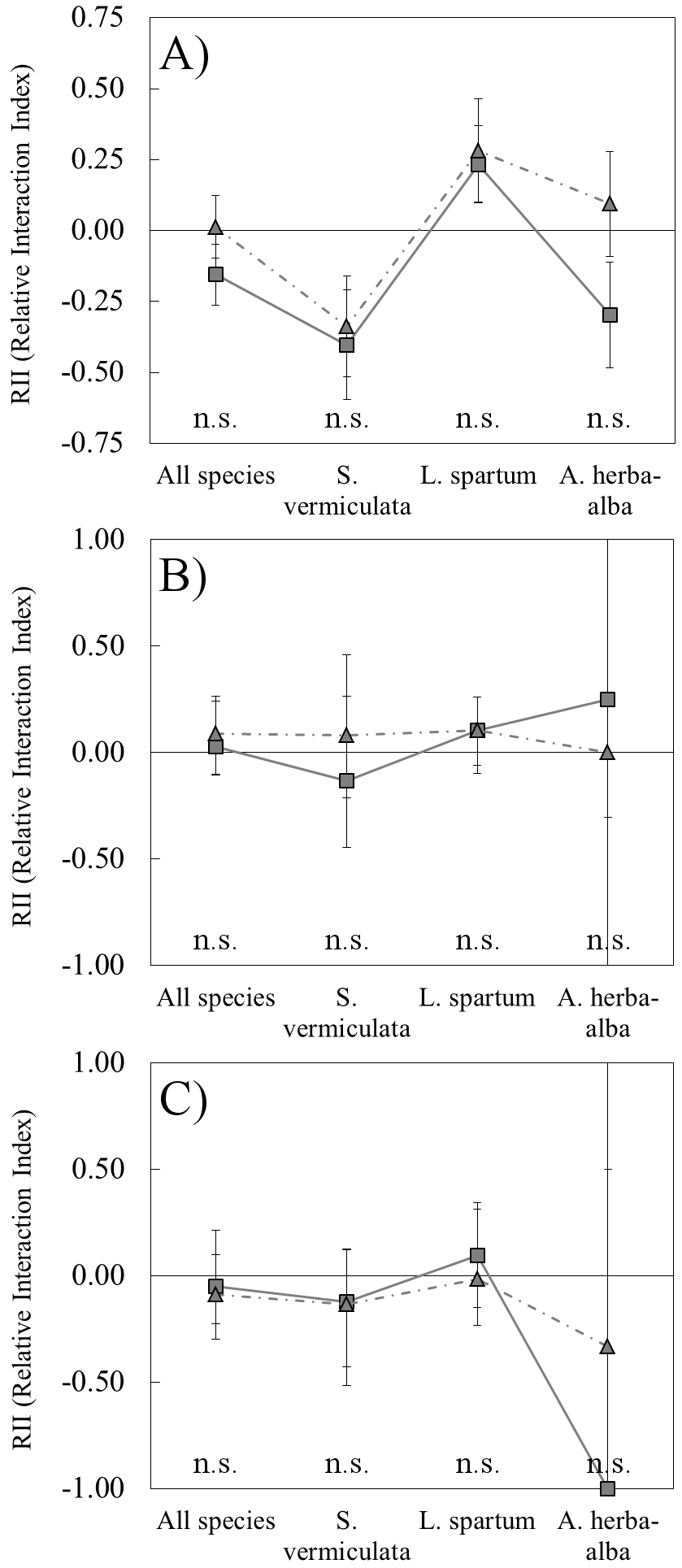

Supplement: S4 Fig — Relative interaction index (RII) calculated for (A) the size (mean ± SE), (B) germination and (C) survival of the target species in A. herba-alba (square symbols) and S. vermiculata (triangle symbols) microsites. Bars in B) and C) indicate the 95% confidence interval obtained using the bootstraping (see material and methods section for further details). n.s., not significant. (TIFF) [file pone.0193421.s004.tiff]
