# Supplementary material for: Evidence for chemical interference effect of an allelopathic plant on neighboring plant species: A field study
Source: PLoS One. 2018 Feb 23;13(2):e0193421. doi: 10.1371/journal.pone.0193421 (PMC5825076; doi:10.1371/journal.pone.0193421)
Supplement: S1 Table — ART, A. herba-alba; SAL, S. vermiculata; BS, bare soil. Significant differences among microsites (ANOVA, p < 0.05) are highlighted in bold. (PDF) [file pone.0193421.s005.pdf]

S1 Table. Initial size (cm^3, mean ± SE) of target species juveniles transplanted by microsite.

	Microsite				
Target species	ART	SAL	BS	F value	*p* (>F)
S. vermiculata	12.68 ± 7.14	26.71 ± 19.43	28.37 ± 20.11	0.267	0.77
L. spartum	246.94 ± 44.57	191.09 ± 79.23	322.75 ± 40.78	1.320	0.28
A. herba-alba	9.11 ± 4.20	37.97 ± 26.54	27.28 ± 15.52	0.664	0.52

ART, *A. herba-alba*; SAL, *S. vermiculata*; BS, bare soil. Significant differences among microsites (ANOVA, $p < 0.05$) are highlighted in bold
